# HIV Neuroinfection and Alzheimer’s Disease: Similarities and Potential Links?

**DOI:** 10.3389/fncel.2018.00307

**Published:** 2018-09-11

**Authors:** Geoffrey Canet, Chloé Dias, Audrey Gabelle, Yannick Simonin, Fabien Gosselet, Nicola Marchi, Alain Makinson, Edouard Tuaillon, Philippe Van de Perre, Laurent Givalois, Sara Salinas

**Affiliations:** ^1^Molecular Mechanisms in Neurodegenerative Dementia, INSERM, University of Montpellier/EPHE, Montpellier, France; ^2^Pathogenesis and Control of Chronic Infections, INSERM, University of Montpellier, Etablissement français du Sang, Montpellier, France; ^3^Memory Research and Resources Center, CHU Montpellier, University of Montpellier, Montpellier, France; ^4^Laboratoire de la Barrière Hémato-Encéphalique, Université d’Artois, Lens, France; ^5^Cerebrovascular Mechanisms of Brain Disorders, Department of Neuroscience, Institute of Functional Genomics, CNRS, INSERM, University of Montpellier, Montpellier, France; ^6^Department of Infectious Diseases CHU Montpellier, INSERM, IRD, University of Montpellier, Montpellier, France; ^7^Pathogenesis and Control of Chronic Infections, INSERM, University of Montpellier, Etablissement français du Sang, CHU Montpellier, Montpellier, France

**Keywords:** HIV-associated neurocognitive disorders, neuroinflammation, viral neuroinfection, Alzheimer’s disease, hypothalamo-pituitary-adrenal axis

## Abstract

Environmental factors such as chemicals, stress and pathogens are now widely believed to play important roles in the onset of some brain diseases, as they are associated with neuronal impairment and acute or chronic inflammation. Alzheimer’s disease (AD) is characterized by progressive synaptic dysfunction and neurodegeneration that ultimately lead to dementia. Neuroinflammation also plays a prominent role in AD and possible links to viruses have been proposed. In particular, the human immunodeficiency virus (HIV) can pass the blood-brain barrier and cause neuronal dysfunction leading to cognitive dysfunctions called HIV-associated neurocognitive disorders (HAND). Similarities between HAND and HIV exist as numerous factors involved in AD such as members of the amyloid and Tau pathways, as well as stress-related pathways or blood brain barrier (BBB) regulators, seem to be modulated by HIV brain infection, leading to the accumulation of amyloid plaques or neurofibrillary tangles (NFT) in some patients. Here, we summarize findings regarding how HIV and some of its proteins such as Tat and gp120 modulate signaling and cellular pathways also impaired in AD, suggesting similarities and convergences of these two pathologies.

## Introduction

Neurodegenerative disorders, whether they are sporadic, genetic or due to pathogens, are caused by the specific impairment (cell death or cell dysfunction) of different neural cell types and represent a major health issue in developed and developing countries. Central nervous system (CNS) impairment can have tremendous effects on day-to-day activities, inducing a loss of autonomy and the need for continuous care and support. Indeed, cognitive processes, as well as locomotor and sensory functions can be severely affected. In this context, the World Health Organization estimated that close to 2 million persons died in 2012 from a neurological disorder (WHO, 2014) and Alzheimer’s disease (AD) International reported that more than 46 millions of people suffered from AD dementia worldwide in 2015 (Prince et al., 2015).

Aging is certainly the primary risk factor for neurodegenerative disorders such as AD and Parkinson’s disease (PD). In AD, the risk doubles every 5 years after the age of 65, and reaches 50% after 85. Genetic factors, often involved in the regulation of neuronal activity or glial function, are also found associated with several types of pathologies such as motor neuron diseases (MND), AD, PD or Huntington’s disease (HD). However, many of these disorders can be sporadic and emphasize the role of the environment in their etiology. For example, in AD, familial forms due to genetic mutations represent less than 5% of cases, whereas sporadic forms are predominant (Selkoe, 2001; Fratiglioni et al., 1993). In these forms multifactorial factors have been identified such as environmental agents that may increase the probability of developing AD (Hayden et al., 2010; Yan et al., 2016). Among such environmental factors, neurotropic infectious agents such as bacteria, parasites or viruses can cause neuronal dysfunction: some pathogens have been selected throughout evolution for their ability to reach the CNS using different strategies such as crossing the blood brain barrier (BBB) or by axonal transport (Smith et al., 2001; Samuel et al., 2007; Salinas et al., 2010; Roe et al., 2014). Once in the CNS, they can trigger a cascade of events directly or indirectly due to their replication cycle, which will lead to neuronal defects, and in some cases, host death. Because some pathophysiological mechanisms involved in neuronal infection and neurodegenerative diseases are relatively similar, and sometimes overlapping, links have been proposed between the onset of certain brain diseases and prior encounters with neurotropic pathogens (Mattson, 2004; De Chiara et al., 2012). For example, some studies proposed potential links between herpes simplex virus (HSV) and/or cytomegalovirus (CMV) infection and the etiology of AD (Itzhaki et al., 1997; Lurain et al., 2013; Itzhaki, 2014). In particular, the presence of HSV in patients carrying susceptibility genes (e.g., *APOE-e4* allele) is associated with the disease (Itzhaki et al., 1997). In the same light, CMV infection has been proposed to have a role in AD by modulating inflammatory responses (Lurain et al., 2013). In this context, accumulating evidence show that neuroinflammation and cerebrovascular permeability are key mechanisms in the etiology of many of these diseases including AD, MND and multiple sclerosis (MS; Hong H. et al., 2016). In the brain, the resident cells involved in inflammation are the glial cells, namely astrocytes and microglia. Interestingly, links between glial-mediated immune response and regulation of cognitive processes are emerging. For example, the pro-inflammatory cytokine tumor necrosis factor tumor necrosis factor-α (TNF-α) can trigger an astrocyte-dependent response that will ultimately lead to excitatory synapses impairment (Habbas et al., 2015). Along the same line, microglia cells are also key regulators of synaptic function and could be responsible for some cognitive deficits after synaptic impairment following activation by viral infections (Vasek et al., 2016), in some brain diseases such as Oculoleptomeningeal amyloidosis (Azevedo et al., 2013) or in AD (Hong S. et al., 2016; Rajendran and Paolicelli, 2018).

One physiological system regulating inflammation is the hypothalamo-pituitary-adrenal (HPA) axis, a major neuroendocrine system. This axis is highly involved in stress responses and triggers the adrenal cortex to release glucocorticoids (GC; cortisol in humans and corticosterone in rodents). These steroid hormones readily cross the BBB and bind to low affinity glucocorticoid receptors (GR) and high affinity mineralocorticoid receptors (MR; Reul and de Kloet, 1985). These receptors are necessary for normal cellular activity, inflammatory and stress responses, and crucial for many CNS functions, including learning and memory (Roozendaal, 2000; Chen et al., 2012). GC via their receptors, increase the transcription of anti-inflammatory genes but also inhibit the expression of multiple inflammatory genes (cytokines, enzymes, receptors and adhesion molecules; Coutinho and Chapman, 2011; Van Bogaert et al., 2011). Interestingly, pathogens can activate the HPA axis and induce subsequent secretion of GC, directly by their structural and genetic components and indirectly by the immune response involving cytokines and inflammatory mediators secreted from activated immune cells and infected tissues (Givalois et al., 1994; Kino, 2000). However, in case of excessive secretion due to chronic stress, GC become unable to exert their effects on target tissues, and trigger the syndrome of GC resistance (Chrousos et al., 1993; Charmandari et al., 2005). Thus, prolonged stimulation of GC secretion can induce or potentiate neuroinflammation, but also excitotoxicity and oxidative stress via synergistic effects with excitatory amino acids such as glutamate (Takahashi et al., 2002; McEwen, 2008).

As yet, there is no definitive and conclusive data showing a causative link between the human immunodeficiency virus (HIV) neuroinfection and the onset of AD. However, accumulating evidence suggests that common pathways and factors are modulated in the brains of HIV^+^ and AD patients, thus pointing out similarities and convergence in these two pathologies. In particular, neuroinflammation is strongly associated with both diseases and is emerging as a major player in the onset and progression of neuropathologies. Understanding the potential link between molecular mechanisms such as neuroinflammation, viral CNS infection, HPA axis deregulation and neurodegenerative disorders is therefore more than pertinent. Here, we summarize existing data, drawing parallels between HIV-associated neurocognitive disorders (HAND) and AD where similar cellular pathways are impaired, and propose potential new links between HIV, HPA pathways and AD that were not previously reported or addressed.

## HIV Neuroinfection

HIV is a retrovirus that depletes CD4^+^ cells and strongly impairs the immune response, thus opening up the way for opportunistic infections that cause the acquired immunodeficiency syndrome (AIDS). However, the immune system is not the only target. HIV is sometimes classified as a neurotropic virus, even though it cannot directly infect neurons, due to their lack of expression of its main receptor CD4 (Peudenier et al., 1991). Nonetheless, the virus can access the brain very early on during primary infection (within the first 2 weeks) where it can locally replicate and become compartmentalized, as determined by recent phylogenetic analyses (Sturdevant et al., 2015). The virus then leads to neurotoxicity that is associated with motor, sensory and cognitive impairment in around 50% of HIV^+^ patients (Becker et al., 2009; Thakur et al., 2018). These neuronal impairments are collectively named HAND (González-Scarano and Martín-García, 2005; Clifford and Ances, 2013; Thakur et al., 2018). Depending on the severity of the symptoms, these conditions are classified into three groups: asymptomatic neurocognitive impairment (ANI), mild neurocognitive disorder (MND) and HIV-associated dementia (HAD) (Antinori et al., 2007). Clinically, patients can display a range of symptoms from cognitive deficits (memory, attention, language, behavior), motor and sensory impairment, mood changes to dementia. Even though asymptomatic, ANI HIV^+^ patients present higher risk for developing cognitive impairments compared to controls and have been proposed to mirror early phases of AD (Ellis et al., 2007; McArthur et al., 2010).

With the introduction of successful combination antiretroviral therapy (cART), the incidence of HAND has decreased (Maschke et al., 2000; González-Scarano and Martín-García, 2005). However, its prevalence is increasing, mainly due to the increased life expectancy of patients, cardiovascular risks factors, exposure hazards, and ongoing nervous system inflammation despite cART. Patients diagnosed with HAND, with mild or severe cognitive impairments, have a lower quality of life and shorter lifespan (Heaton et al., 2011). Before the establishment of cART, HAD could be found in up to 15%–20% of HIV^+^ individuals and was one of the main risk factor (McArthur et al., 1993, 2003). In the post-cART era, the total proportion of patients with HAND did not vary but the distribution of the classes changed with a decrease in HAD and an increase in MND and ANI (González-Scarano and Martín-García, 2005). Moreover, neuronal disorders are becoming more frequent in the aging HIV^+^ population (Thakur et al., 2018). Prospective trials have found poor prediction on the effect of cART on the patient’s cognitive impairment as the BBB limits the penetration of the drugs into the brain and some antiretrovirals (ARV) can show neurotoxicity (Soontornniyomkij et al., 2018; Thakur et al., 2018). In this context, HAD has also been proposed as the most common form of dementia in people under 40 years of age (Janssen, 1992).

**Figure 1:**
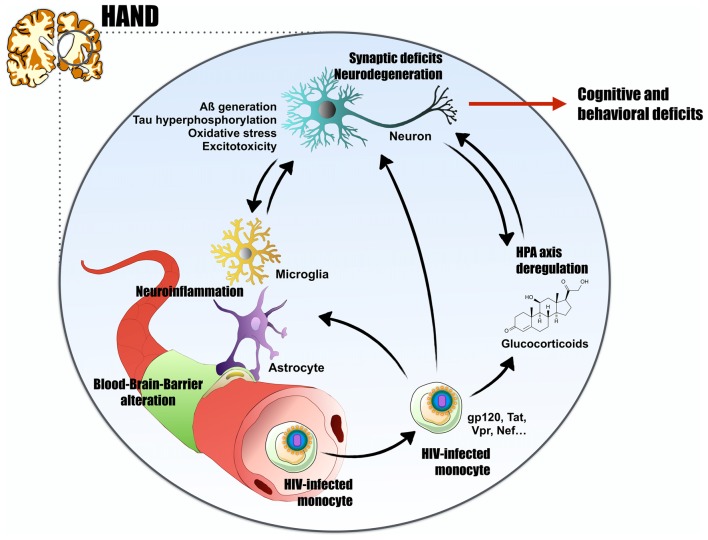
Proposed mechanism for human immunodeficiency virus (HIV)-associated neurocognitive disorders (HAND). HIV-1, through infected monocytes, can cross the blood brain barrier (BBB) by the Trojan horse mechanism. In the central nervous system (CNS), neuroinflammation triggered directly by viral replication or by HIV-viral proteins (glycoprotein 120 (gp120), transactivator of transcription (Tat), viral protein R (Vpr), negative regulatory factor (Nef)…) exert neurotoxic effects. They impact neurons integrity and lead to Alzheimer’s disease (AD)-like characteristics such as A generation, abnormal Tau phosphorylation, oxidative stress and excitotoxicity. The virus can induces neuroinflammation by several mechanisms: direct infection of astrocytes, BBB impairment and peripheral macrophages invasion, or massive gliosis and cytokines release. Finally, HIV^+^ patients present high glucocorticoids (cortisol) levels, characteristic of a hypothalamic–pituitary–adrenal (HPA) axis deregulation. Glucocorticoids and their receptors are highly involved in the etiology of AD. By these numerous pathways, HIV-1 induces synaptic deficits and neurodegeneration, thus leads to cognitive and behavioral deficits, and could explain the establishment of HAND in HIV^+^ patients and potentially the onset of AD.

HIV enters the CNS by a mechanism called “Trojan horse” that consists of the migration of infected monocytes through the BBB (Williams et al., 2015; Zhang et al., 2015). Recent work showed that CD14^+^CD16^+^ monocytes are able to efficiently transmigrate through the BBB and are found in high numbers in HIV-infected individuals (Williams et al., 2015). Once in the brain, HIV can infect various cell types expressing the CD4 receptor including microglia (Cosenza et al., 2002), perivascular macrophages and potentially adult neural precursors (Rothenaigner et al., 2007). Viral replication is also seen in a restrictive manner in astrocytes (Eugenin et al., 2011). These observations have led to the classification of the brain as a reservoir and a sanctuary for HIV (Hellmuth et al., 2015). In the brain, direct and indirect effects of HIV infection will lead to neuronal dysfunction (Figure 1): infected astrocytes and microglia release factors inducing neurotoxicity such as cytokines, chemokine and reactive oxygen species (ROS) (González-Scarano and Martín-García, 2005). Later, this may also contribute to the disruption of the BBB and result in further entry/exit of virions and viral proteins (see below). Some HIV proteins (glycoprotein120 (gp120), transactivator of transcription (Tat), viral protein R (Vpr) and negative regulatory factor (Nef)) can have direct effects on neurons and trigger signaling cascades leading to neuronal impairment. These proteins can be released from infected non-neuronal cells or shed from virions (Nath, 2002; Churchill et al., 2015). Consistently, some viral proteins such as Tat and Vpr are found in the cerebrospinal fluid (CSF; Levy et al., 1994; Hudson et al., 2000; Nath, 2002). Numerous studies using *ex vivo* and *in vivo* models show a plethora of neurotoxic effects: the envelope protein gp120 was shown to promote the release of IL-1β and TNFα, as well as neurotoxic factors such as glutamate, which in turn triggers neuronal apoptosis. (Garden et al., 2002; Bachis et al., 2009). Tat potentiates glutamate overactivation of NMDA receptors and release of cytokines through astrocytes (Haughey et al., 2001; King et al., 2006) and triggers neuronal apoptosis (Kruman et al., 1998). Tat and gp120-mediated apoptosis also triggers an increase in intracellular calcium levels which is classically associated with excitotoxicity processes and is induced by glutamate accumulation in the extracellular space. HIV^+^ patients show elevated CSF glutamate levels that correlate with dementia severity and the degree of brain atrophy (Ferrarese et al., 2001). Similarly, the protein Nef can trigger cytotoxic effects (Sami Saribas et al., 2017). Vpr has been proposed to induce mitochondrial neuronal accumulation and impaired axonal transport by modulating microtubule stability (Wang et al., 2017).

Ultimately, if the viral load is not controlled, neuronal loss will occur. Interestingly, similarly as during the early stages of AD (Bateman et al., 2012; Marcello et al., 2012), HIV-induced neurodegeneration does not correlate completely with cognitive impairments. Cognitive deficits in patients with HAD have been shown to better correlate with synaptic impairment than neurodegeneration as cell death is also accompanied by axonal degeneration, astrocytosis and synaptic loss (Adle-Biassette et al., 1999; Avdoshina et al., 2013). Moreover, some studies aimed to decipher whether neurocognitive deficits are still found in populations where the viral load is well controlled. While some cohorts showed that cognitive functions are generally not affected in individuals with no detectable viremia (Lopardo et al., 2009), others suggest that there is still a high prevalence of HAND in HIV^+^ aviremic patients (Cysique et al., 2006; McArthur et al., 2010). This could be explained by several non-exclusive mechanisms such as: (i) poor cART regime penetration of the BBB; (ii) toxicity of anti-retrovirals; (iii) prolonged lifespan of infected individuals; (iv) local low-noise viral replication; and (v) chronic neuroinflammation. Continuous inflammatory injuries could pave the way for some neurodegenerative disorders. Potential links have therefore been suggested between HIV and amyotrophic lateral sclerosis (ALS; Alfahad and Nath, 2013) and AD (Chakradhar, 2018; see below).

## Key Features of AD

AD is a devastating neurodegenerative disorder and the most common cause of neurodegenerative dementia in the elderly. It is characterized by a progressive impairment of cognitive functions, associated with synaptic and neuronal loss, as well as senile plaques and neurofibrillary tangles (NFT) in the brain (Selkoe, 2001; Cummings, 2004). Plaques are composed of insoluble extracellular aggregates consisting mainly of amyloid-β (Aβ) peptides, whereas NFT are the result of hyper- and abnormal phosphorylation on the intracellular microtubule-stabilizing protein Tau (Selkoe, 2001). Aβ production is dependent of the amyloidogenic pathway induction, and results from a pathological cleavage of the amyloid precursor protein (APP) by β-secretase (BACE1), which releases the C99 fragment, and a second cleavage by γ-secretase releasing Aβ into the extracellular medium (Cummings, 2004; Flammang et al., 2012). This generation of Aβ, in synergy with Tau, induces a cascade of neurodegenerative mechanisms such as cholinergic neurons degeneration, synaptic deficit, neuroinflammation, oxidative stress, apoptosis and autophagy, in part responsible for cognitive and behavioral deficits (Selkoe, 2001; Cummings, 2004). Moreover, neurotoxicity in AD is correlated with the total level of brain circulating oligomeric Aβ peptides, mainly consisting of Aβ_1–40_ and Aβ_1–42_ peptides (Selkoe, 2001; Cummings, 2004; Cleary et al., 2005; Blennow et al., 2010).

## Common Mechanisms Between HIV Neuroinfection and AD

Because AIDS became a chronic disease with the advent of cART, an important proportion of HIV^+^ patient population is now over 50 years of age and also facing age-related disorders (Milanini and Valcour, 2017). In addition, accelerated ageing, including immunosenescence, is a constitutive part of the natural history of HIV infection. In particular, HAND is weakening an already age-targeted organ and could favor the occurrence of neurodegenerative diseases. Regarding AD, questions have risen about potential links with HIV CNS infection from observations and studies demonstrating modulation of common pathways/mechanism (such as the amyloid and Tau pathways). In this context, several symptoms associated with AD physiopathology were reported in HIV^+^ patients and murine *in vivo* and *in vitro* models of neuroAIDS (Table 1).

**Table 1:** Common neurotoxicity pathways between Alzheimer’s disease (AD) and human immunodeficiency virus (HIV).

AD symptoms	Observation in HIV^+^ patients	Observations in *in vivo* and *in vitro* HIV models
Misprocessing of APP and Aβ synthesis	Increase in CSF Aβ_1–42_ (ELISA; Brew et al., 2005) Presence of amyloid plaques in brain (Esiri et al., 1998; Green et al., 2005)	Increase of amyloid plaques production (Congo red staining; Cho et al., 2017) Increase of C99 fragment (western blot; Cho et al., 2017) Increase of Aβ_1–42_ (ELISA; Kim et al., 2013)
Abnormal Tau phosphorylation	Increase in CSF total and phosphorylated Tau (ELISA; Brew et al., 2005; Anthony et al., 2006) Increase of GSK3β, Cdk5 and p35 in frontal cortex (Patrick et al., 2011)	Increase of p-Thr181, p-Thr231, p-Ser396, p-Ser404 (western blot; Kang et al., 2010; Cho et al., 2017) Increase of Cdk5 and GSK3β contents (western blot; Kang et al., 2010; Cho et al., 2017)
Activation of death pathways and apoptosis	Increase of apoptosis (TUNEL; Lannuzel et al., 1997) Increase of JNK/ERK contents and activities (western blot and kinases assay; Lannuzel et al., 1997)	Increase of caspase 3, Bax, pJNK/JNK, Erk contents (western blot; Kruman et al., 1998; Kaul and Lipton, 1999; Cho et al., 2017) Increase of apoptosis (TUNEL; Kruman et al., 1998)
Oxidative Stress	Oxidative stress, ROS production, mitochondrial dysfunction, impaired glucose metabolism (Vignoli et al., 2000)	Increase of NAPDH oxydase, CYP2E1, iNOS, IkB, HIF-1 (western blot; Cho et al., 2017)
Neuroinflammation	Massive gliosis through peripheral macrophages invasion and chemokines release (Peters et al., 2004)	Increase of astrocytes GFAP and microglial Iba1 (western blot and histology; Kang et al., 2010; Cho et al., 2017) Increase of proinflammatory cytokines (TNF, IL6, MCP-1; western blot; Cho et al., 2017)
Excitotoxicity	Increased CSF glutamate levels (ELISA; Ferrarese et al., 2001)	Increase glutamate release and decrease glutamate reuptake by astrocytes (Dreyer and Lipton, 1995; Belmadani et al., 2001)
Neurodegeneration	Cortical gray and white matter loss (post mortem histological study; Masliah et al., 1992) About 20%–50% neuronal loss in the frontal cortex (Ketzler et al., 1990)	Decrease of NeuN (western blot; Kang et al., 2010; Cho et al., 2017) Impaired neurogenesis (Mishra et al., 2010)
Cognitive and learning deficits	Decrease of memory performances (Becker et al., 2009)	Learning deficits (Morris Water Maze; Vigorito et al., 2007)
Blood Brain Barrier	Increased CSF/plasma albumin ratio in HAD patients (Anesten et al., 2016)	HIV infection increases leukocytes transmigration through tight junctions (TJs) proteins down regulation and metalloproteinases upregulation (Eugenin et al., 2011)
HPA axis deregulation	Glucocorticoid resistance, modification of glucocorticoid sensitivity, altered cytokine production (Chrousos and Zapanti, 2014) Adrenal insufficiency, elevated plasma GC (Christeff et al., 1997; Kino, 2000)	Increase of hypothalamic CRF levels, AVP levels and CRF mRNA levels (Costa et al., 2000)

HIV^+^ patients presenting HAND show CSF features very similar to early and late stages of AD (Table 1). For instance, biomarkers such as Aβ_1–42_ are found to be dysregulated in the CSF of HAND patients (Clifford et al., 2009). When comparing CSF from age-matched controls, HAND and late-stage AD patients, a similar trend for decreased Aβ_1–42_ levels was found in HIV^+^ individuals suffering from neuronal disorders (522 pg/ml for HAND and 421 pg/ml for AD, compared to 722 pg/ml for controls; Clifford et al., 2009). Notably, HIV^+^ patients without neurological symptoms had levels of Aβ_1–42_ in the same range as non-dementia controls (Clifford et al., 2009). Consequently, plaques caused by extracellular amyloid peptide accumulation can be seen in patients, particularly before the cART era (Esiri et al., 1998; Green et al., 2005).

Emerging evidence suggests that brain exposure to HIV particles and HIV proteins can directly or indirectly modulate the amyloid and Tau pathways (Chen et al., 2013; Ortega and Ances, 2014; Cho et al., 2017; Hategan et al., 2017). In HIV murine models (HIV-1 transgenic rats and gp120 transgenic mice) neurodegeneration is observed and associated with apoptosis, gliosis, oxidative stress, Aβ synthesis and increases in Tau phosphorylation (Table 1). Tat can affect Aβ synthesis through several mechanisms. It can increases Aβ production by modulating endolysosomal structure and function (Chen et al., 2013). Conversely, Tat leads to an increase of Aβ accumulation by inhibiting its degradation by neprilysin (Daily et al., 2006), and increasing BACE1 activity and synthesis of the C99 fragment (Chen et al., 2013; Cho et al., 2017). This increase in BACE1, which is also found elevated in AD, was recently confirmed in HIV^+^ patients (Stern et al., 2018). Similarly, treatment of primary hippocampal cell cultures with recombinant gp120 promotes Aβ_1–42_ secretion (Aksenov et al., 2010). In addition, when lentiviral vector-derived Tat is expressed in the hippocampus of APP/PS1 transgenic mice (a widely used mouse model of AD), it potentiates Aβ_1–42_ synthesis and increases the size of amyloid plaques (Kim et al., 2013). More recently, it was demonstrated that in primary hippocampal neurons, Tat interacts with Aβ peptides and forms complexes that increase damage likely through membrane pore formation (Hategan et al., 2017). In HIV-1 transgenic rats (which express seven of the nine HIV-1 viral proteins including gp120, Nef and Tat), the number and size of amyloid plaques were significantly elevated in the cerebral cortex compared to wild type (WT) animals (Cho et al., 2017). This was accompanied by an increase of amyloid C-terminal fragment C99 levels (>5-fold) in brains of HIV-1 transgenic rats (Cho et al., 2017). Similarly, The HIV-1 matrix protein p17 released from HIV-1 infected cells, participates in amyloid deposits toxicity by its ability to misfold and aggregate, even in the presence of protease inhibitors (Zeinolabediny et al., 2017). When injected into the mouse hippocampus, p17 colocalizes with phospho-Tau, plaque and fibril-like structures, where it increases Aβ expression and plaque-like development (Zeinolabediny et al., 2017). This led to cognitive impairment, as measured by recognition and Morris water maze tests (Zeinolabediny et al., 2017). Recently, APP metabolism in macrophage and microglia was reported to be modulated by HIV-1 Gag protein (Chai et al., 2017). The Gag polyprotein was shown to increase Aβ production and associated neurotoxicity by the activation of secretases. APP on the other hand, was reported to act as an antiviral factor by sequestering Gag in lipid rafts and restricting HIV-1 release (Chai et al., 2017). The balance between these two mechanisms (restriction and evasion), as well as the impact on Aβ peptide genesis will need further investigation.

The causative role of Tau and/or hyperphosphorylation of Tau in HAND is still poorly established. However, in 10 month-old gp120 transgenic mice, cognitive abnormalities, which are associated with an increase in neuronal death and gliosis, are also linked with an increase in Tau hyperphosphorylation (Kang et al., 2010). This effect is concomitant with an over-activation of GSK3-β, the main enzyme involved in Tau phosphorylation (Kang et al., 2010). In HIV-1 transgenic rats, levels of phosphorylated-Tau (p-Thr181, p-Thr231 and p-Ser396) were markedly elevated in the hippocampus and associated with an increase in Cdk5 activity, the other main enzyme involved in Tau phosphorylation (Cho et al., 2017). These animal model observations are in accordance with significantly elevated levels of phospho-Tau and abnormal NFT in HIV^+^ patients with HAND (Brew et al., 2005; Anthony et al., 2006; Kang et al., 2010).

## HIV, AD and the Blood Brain Barrier

Neurodegenerative disorders, including HAND, are often associated with BBB impairment (Atluri et al., 2015; Zhao et al., 2015). Endothelial microvascular cells, pericytes, neurons and astrocytes form and regulate the BBB neurovascular unit (NVU), a tightly regulated endothelium that separates the brain from the systemic circulation. The BBB is also a metabolic barrier because endothelial cells express several enzymes and efflux pumps impeding the entry of xenobiotics and cells into the CNS (Cecchelli et al., 2014). When the modulation of the microenvironment by inflammation or cell damage occurs, BBB integrity can be perturbed. In AD, the damage of microvessels is believed to be associated with the progression of the disease (Johnson et al., 2005; Marchesi, 2011; Rosenberg, 2014). In mouse models and in AD patients, an alteration of the BBB physiology exists and is linked with amyloid deposits (Gosselet et al., 2013; Montagne et al., 2017; Yamazaki and Kanekiyo, 2017) Moreover, Aβ peptides are normally cleared from the brain by specific transport across the BBB. Receptors and transporters expressed by NVU cells like LRP1, the P-glycoprotein (P-gp) and Breast Cancer Resistance Protein (BCRP), play an important role in Aβ transport through the BBB (Gosselet et al., 2013; Storck et al., 2016). LRP1 may also be involved in Aβ endocytosis in endothelial cells for degradation via the lysosomal pathway (Nazer et al., 2008). Other receptors such as the receptor for advanced glycation end products (RAGE) are involved in Aβ entry into the CNS (Candela et al., 2010; Bu et al., 2017). Downregulations of LRP1, P-gp and BCRP or upregulation of RAGE during AD potentiate the Aβ accumulation (Gosselet et al., 2013; Yamazaki and Kanekiyo, 2017). In addition, Aβ can lead to a decrease of tight junction (TJ) proteins (occludin, claudin-5 and ZO-1) in endothelial cells and reduction of microvessel coverage by pericytes, which induces higher BBB permeability (Gosselet et al., 2013; Yamazaki and Kanekiyo, 2017).

During HIV-1 infection, impairment of the BBB occurs and is probably responsible for the spread of virions from the vascular compartment, enhances immune cell recruitment, and may allow brain infection by other opportunistic pathogens (Atluri et al., 2015; Zhang et al., 2015; Anesten et al., 2016). The interaction between HIV-1 and the BBB occurs with all of the NVU cells and often involves viral proteins. HIV-infected astrocytes can directly impair BBB integrity by dysregulating gap junctions (Eugenin et al., 2011). Tat, gp120, Vpr and Nef, have shown cellular alteration, dysregulation of molecular pathways and an inhibition of repair mechanisms resulting in impairment of the BBB (Zhang et al., 2015). Tat has been shown to directly modulate the endothelium through several cellular pathways, including the inhibition of the RhoA/ROCK or Ras pathways, which lead to the downregulation of TJ proteins expression, resulting in the BBB impairment (Chen et al., 2012, 2016; Jiang et al., 2017). These effects are accompanied by an accumulation of Aβ peptide in the brain, suggesting a direct role for HIV proteins in Aβ-BBB interaction. Interestingly, Tat can also modulate the expression of receptors and transporters of Aβ, which are involved in bi-directional transport of peptides across the BBB. Extracellular Tat can upregulate RAGE expression resulting in Ras/MAPK signaling pathway activation and *in fine* Aβ accumulation (András and Toborek, 2013; Chen et al., 2016). Tat can also reduce Aβ clearance across endothelial cells in inhibiting the synthesis of LRP-1 (Chen et al., 2016). Similarly, gp120 has been shown to impair BBB integrity through the PKC and JAK/STAT pathways and to enhance monocyte migration, a process that can also increase the number of HIV-infected monocytes that cross the BBB to get to the CNS (Kanmogne et al., 2007; Yang et al., 2009; Zhang et al., 2015). Conversely, rats injected with recombinant gp120 in the caudate putamen showed lesion in brain microvessels, suggesting that gp120 could directly impair brain endothelial cell physiology and therefore BBB integrity (Louboutin et al., 2010).

Ultimately, these mechanisms could disturb Aβ clearance through the interstitial fluid, resulting in an increase in Aβ deposit and accumulation. In this context, it is more than pertinent to consider the role of the BBB in AD and HAND pathogenesis (Yamazaki and Kanekiyo, 2017).

## HIV, HPA Axis Dysregulation and AD: GC as a Link Between HIV Infection and AD?

In HIV^+^ patients treated with cART, the adrenal gland is frequently affected, resulting in higher serum cortisol levels both in early stages and severely affected patients (Sellmeyer and Grunfeld, 1996; Christeff et al., 1997; Collazos et al., 2003; Langerak et al., 2015). In addition, an excess of circulating GC can also dysregulate numerous brain functions. As GC act in synergy with glutamate, a deregulation of the HPA axis activity or a modification of GR function can be toxic, especially in the hippocampus by inducing excitotoxicity, neuronal damage (neuronal death by apoptosis and synaptic deficits), neuroinflammation, oxidative stress and cognitive decline (Magariños and McEwen, 1995; de Kloet et al., 2005). In fact, it appears that plasma cortisol levels correlate with the severity of hippocampal atrophy and therefore could contribute to the cognitive decline and psychological symptoms that occur in neurodegenerative pathologies and particularly in AD (Lupien et al., 1998). In AD, this view is particularly sustained by the fact that cognitive deficits and psychological symptoms are associated with an early deregulation of the HPA axis, as well as elevated levels of GC in plasma and CSF (Csernansky et al., 2006; Hoogendijk et al., 2006). Moreover, GC and GR can directly trigger APP misprocessing and Aβ pathway through the direct transcription of APP and BACE1 genes (Lahiri, 2004). Along this line, GC and stress appear to induce abnormal Tau hyperphosphorylation and accumulation (Sotiropoulos et al., 2011). This suggests that dysregulations of the HPA axis would likely increase Aβ pathology and subsequent Tau accumulation and hyperphosphorylation, resulting inexorably in a vicious circle whereby the pathology increases the secretion of GC, which further increases the pathology (Green et al., 2006; Brureau et al., 2013; Pineau et al., 2016).

**Figure 2:**
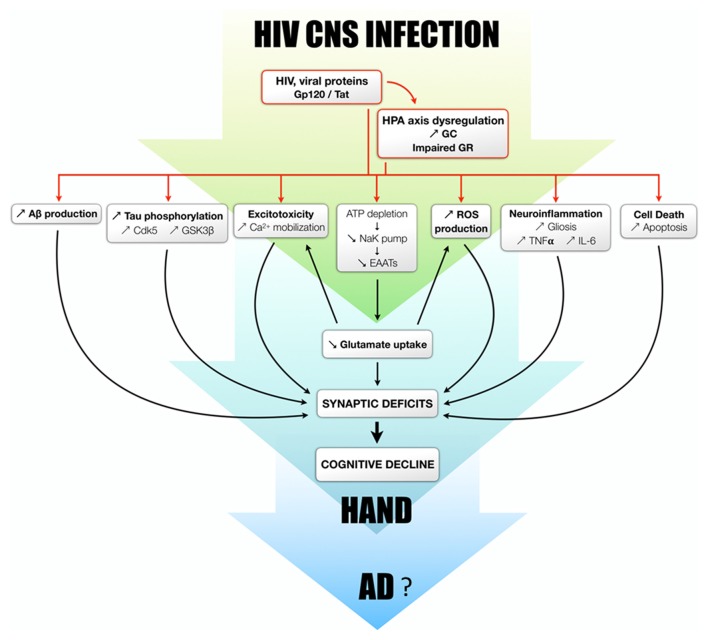
Proposed mechanisms by which glucocorticoids and their receptors modulate/potentiate the development of HAND and potentially AD. The dysregulation of the HPA axis is observed both in HIV patients and rodent models. GC overexposure, in combination with viral proteins or not, is able to induce the increase of Aβ production, Tau phosphorylation, excitotoxicity, oxidative stress, neuroinflammation and apoptosis. It should be also mentioned that Aβ itself can trigger Tau phosphorylation, excitotoxicity, oxidative stress, neuroinflammation and cell death. All these processes lead to neurodegeneration and synaptic deficits and potentially responsible for cognitive decline observed in HAND patients, all of which could progressively favor to the development of AD.

In HAND, viral proteins such as Tat and gp120, together with GC, could have synergistic effects on excitotoxicity and oxidative stress, by decreasing glutamate uptake by different but complementary processes, as previously mentioned. This is illustrated by the fact that combined treatment of GC and gp120 directly increases calcium mobilization, ATP depletion, decrease of mitochondrial potential, ROS production and neurotoxicity (Brooke and Sapolsky, 2003). Basal levels of GC enhanced the disruptive effects of gp120 on metabolism in the hippocampus and in the cortex. Moreover, raising GC concentration exacerbated the ability of gp120 to mobilize cytosolic calcium in hippocampal cells (Yusim et al., 2000; Brooke and Sapolsky, 2003). Acute exposure to GC initiated the endocytosis of glucose transporter in neurons and glia, whereas chronic exposure to GC could directly inhibit its transcription, resulting in energy depletion (Brooke and Sapolsky, 2003). The decrease of glucose transporter is responsible for a lack of ATP production, necessary to supply sodium/potassium pumps that are part of the glutamate transporter system (EAATs; Virgin et al., 1991; Chan et al., 1996). Gp120 and Tat act on astrocyte, microglia and neurons. Especially, gp120 induces arachidonic acid and superoxide release that in turn inhibits glutamate uptake by glia cells (Dreyer and Lipton, 1995; Belmadani et al., 2001). So, taken together, GC, Tat and gp120 strongly block glutamate uptake (Wang et al., 2004; Cheung et al., 2008; Potter et al., 2013), which could potentiate excitotoxicity and neuronal loss observed in HAND patients. Such mechanisms were already described in AD, and potentially due to GC excess (Virgin et al., 1991; Goodman et al., 1996). Even if this view is speculative and needs further investigation, GC and HPA axis deregulation could participate in neuronal dysfunction in HIV patients. Excessive GC secretion, in synergy with viral proteins, could trigger and/or accelerate the onset of the pathology by altering APP processing, Tau phosphorylation and BBB integrity. In addition, synergetic action between GC and HIV could potentiate oxidative stress induction, excitotoxicity and neuroinflammation and, finally, cognitive decline (Figure 2).

## Perspectives and Conclusion

In recent years, a consensus has emerged regarding the etiology of neurological disorders, which is now believed to be complex and multifactorial, even in the case of familial (genetic) forms. A concept that is clearly emerging though is that acute and chronic (neuro) inflammation may pave the way to such disorders and neurotropic pathogens may therefore represent likely candidates among environmental factors promoting this inflammatory/causative state. Inflammation, in particular in the brain, is a clear hallmark of HIV infection, and can also be found in virally suppressed individuals (Edén et al., 2007). *in vitro* and *in vivo* studies show neurotoxic effects of some HIV proteins. Direct and indirect effects, coupled to the potential toxicity of some anti-HIV drugs could provide a weakened environment where the onset of neurological disorders (e.g., AD), could be favored.

HIV neuroinfection shares many pathways and characteristics of familial and sporadic forms of AD, but whether HIV patients will have more chance to develop bona fide AD is not yet fully understood and will have to be determined in the following years. The HIV^+^ population is aging and starting to face age-related disorders. However, there is still little information on their susceptibility to neuronal disorders besides what qualifies as HAND. Although large epidemiological studies are still needed to definitively conclude that aging HIV^+^ patients are more at risk of developing AD, there are however key physiological findings that suggest that this could be the case, though debate between neurologists exists (Ortega and Ances, 2014; Chakradhar, 2018). In this context, it was reported that HIV-1 modulates Aβ metabolism differently from what is observed in AD (in terms of Aβ deposition patterns), which suggests unique features for HAD and HAND (Ortega and Ances, 2014). This also suggests that the onset of dementia in aged HIV^+^ patients could be due to either HIV but also to AD or a combination of both. Identification of HIV^+^ patients with AD are starting to be reported and may provide more clinical data regarding how these two diseases may or may not be connected (Mäkitalo et al., 2015; Turner et al., 2016). Because of the similarities between HAND and AD one of the challenges facing neurologists will be to discriminate between advanced cognitive impairment caused by HIV (HAD) and AD (Xu and Ikezu, 2009). Distinctive CSF biomarker profiles, coupled to appropriate imaging techniques are keys to understanding and diagnosing these pathologies. In this light, the study by Mäkitalo et al. (2015) is of particular importance as it reports the CSF pattern of a HIV^+^ patient having developed AD, possibly because of HIV CSF escape.

In conclusion, the causes behind sporadic forms of AD are still poorly characterized, but environmental factors, in combination or not with genetic triggers, are emerging as key players in their onset. In particular, the dysregulation of the HPA axis that can associated with HIV infection could favor an environment where oxidative stress, neuroinflammation, excitotoxicity, BBB disruption and amyloid-β load are exacerbated and thus, combined with other (genetic or environmental) factors pave the way for the establishment of brain diseases such as AD. It is therefore not unlikely that HIV infection may represent a risk factor for AD and other related neuronal disorders.

## Author Contributions

SS and LG participated in manuscript design, writing and coordination. GC, CD, AG, YS, FG, NM, AM, ET and PP participated in manuscript writing and editing. All authors read and approved the final manuscript.

## Conflict of Interest Statement

The authors declare that the research was conducted in the absence of any commercial or financial relationships that could be construed as a potential conflict of interest.

## Abbreviations

AD: Alzheimer’s disease
AIDS: acquired immunodeficiency syndrome
ALCAM: activated leukocyte cell adhesion molecule
ALS: amyotrophic lateral sclerosis
ANI: asymptomatic neurocognitive impairment
APP: amyloid precursor protein
Aβ: amyloid-β peptides
BACE1: β-secretase converting enzyme 1
BBB: blood brain barrier
BCRP: breast cancer resistance protein
cART: combination antiretroviral therapy
CMV: cytomegalovirus
CNS: central nervous system
CSF: cerebrospinal fluid
GC: glucocorticoids
gp120: glycoprotein 120
GR: glucocorticoid receptors
HAD: HIV-associated dementia
HAND: HIV-associated neurocognitive disorders
HD: Huntington disease
HIV: human immunodeficiency virus
HPA axis: hypothalamo-pituitary-adrenal axis
HSV: Herpes simplex virus
JAM-A: junctional adhesion molecule A
LPR: low-density lipoprotein receptor-related protein
MND: mild neurocognitive disorder
MND: motor neuron diseases
MR: mineralocorticoid receptors
MS: multiple sclerosis
Nef: negative regulatory factor
NFT: neurofibrillary tangles
NVU: neurovascular unit
P-gp: P-glycoprotein
PD: Parkinson’s disease
RAGE: receptor for advanced glycation end products
ROS: reactive oxygen species
Tat: transactivator of transcription
TJ: tight junctions
TNF-α: tumor necrosis factor-α
Vpr: viral protein R.
